# In-laboratory breast specimen radiography reduces tissue block utilization and improves turnaround time of pathologic examination

**DOI:** 10.1186/s12880-021-00589-1

**Published:** 2021-03-23

**Authors:** Sri Krishna Chaitanya Arudra, Laura C. Garvey, Ian S. Hagemann

**Affiliations:** Department of Pathology and Immunology, Washington University School of Medicine, 425 S. Euclid Ave., Campus Box 8118, St. Louis, MO 63110 USA

**Keywords:** Breast, Surgical pathology, Radiography, Quality improvement, Diagnostic tests, Faxitron

## Abstract

**Background:**

This study was performed to determine whether in-laboratory specimen radiography reduces turnaround time or block utilization in surgical pathology.

**Methods:**

Specimens processed during a 48-day trial of an in-lab cabinet radiography device (Faxitron) were compared to a control group of specimens imaged in the mammography suite during a prior 1-year period, and to a second group of specimens not undergoing imaging of any type.

**Results:**

Cases imaged in the mammography suite had longer turnaround time than cases not requiring imaging (by 1.15 days for core biopsies, and 1.73 days for mastectomies; p < 0.0001). In contrast, cases imaged in-lab had turnaround time that was no longer than unimaged cases (p > 0.05 for core biopsies, lumpectomies and mastectomies). Mastectomies imaged in-lab required submission of fewer blocks than controls not undergoing any imaging (mean reduction of 10.6 blocks).

**Conclusions:**

Availability of in-lab radiography resulted in clinically meaningful improvements in turnaround time and economically meaningful reductions in block utilization.

## Background

Radiology-pathology correlation plays an important role in several aspects of breast pathology [1]. In particular, presurgical evaluation often identifies microcalcifications as areas of interest, and/or results in placement of radiopaque clips as fiducial markers for future pathologic examination.

Microcalcifications are associated with a variety of benign and malignant breast diseases [2], and if sufficiently suspicious may be an indication for biopsy [3]. When a biopsy or excision is performed for microcalcifications, corresponding calcifications must be described in the resulting specimen. If absent, serial examination of deeper levels is typically required until either the calcifications are found or the block is exhausted [4]. Failure to identify the calcifications may result in malignant specimens being misclassified as benign [5].

In the case of a core biopsy, where the number of tissue blocks is usually small (often < 5), it is feasible to prepare deeper sections on all of the blocks, but if some of the blocks do not harbor calcifications, the effort to cut additional levels is wasted. In the case of resection specimens (partial or total mastectomy), the total number of blocks is likely to be much larger, and step sectioning of the blocks becomes impractical. In examining breast excision specimens, it is also common to embed only a fraction of the total tissue. If calcifications are not identified histologically, it may not be clear whether the calcifications are present deeper in the existing blocks, or are still in the tissue not yet embedded. This concern persists even if some calcifications are identified in blocks of a subtotally embedded excision specimen.

In current practice, radiopaque metal clips are often placed in the breast at the time of core biopsy, in order to aid in subsequent identification of the site of interest. Matching these clips to those present in the surgical specimen allow the pathologist to document that the site of prior biopsy has been excised. Various clip shapes (barrel-shaped, wing-shaped, rod-shaped, etc.) exist, making it possible to differentiate biopsy sites from one another. Radiofrequency identification devices have also been described for this purpose [6]. Gross examination of breast excision specimens therefore often focuses on localization of the clip or clips, so that the surrounding tissue can be embedded for histologic examination.

When clips are not grossly identified, a larger than usual amount of tissue may be selected for embedding, either in the hope of finding histologic evidence of a small or lost clip in grossly unremarkable tissue, or on the assumption that the larger volume of examined tissue will encompass the area of interest. Difficulty in locating clips is also responsible for much of the time required for prosection of breast specimens. Even when clips are ultimately identified, locating them can require significant time (potentially > 1 h for processing of a single specimen). Meticulous sectioning of tissue results in specimen disruption and degradation, makes it difficult to determine the size of lesions, and can separate lesions from the closest margin. There may be controversy about the number or shape of clips included in the specimen.

A useful approach to “missing” calcifications is to acquire radiographs of breast tissue cores, core biopsy paraffin blocks, breast excision blocks, or entire excision specimens either before or after initial prosection. This can be done in a protocolized way (e.g., all core biopsy specimens radiographed prior to embedding), or tailored to each case. The latter approach may ensure consistency but increases cost, as many of the radiographs will not be needed. The large number of different scenarios that can arise makes it unlikely that any single protocol can cover all eventualities (e.g., if all total mastectomies are imaged prior to prosection, there may still be cases that require imaging at a later time, for example if clips cannot be identified after submission of a large number of blocks).

Another parameter is whether the radiography is done in the radiology suite on equipment shared with clinical patient imaging, versus on dedicated equipment present in the pathology department. Advantages of the former approach include that it may not require additional equipment purchases, and that it may facilitate interpretation of the images by board-certified radiologists. Disadvantages include the opportunity cost of using clinical equipment to image specimens, since during that time it cannot be used to image patients. It is likely that imaging of pathologic specimens, which are relatively small, does not require a full mammography workstation. Also, since clips are conspicuous by imaging, radiologist professional time may not be required.

Use of in-lab specimen radiography has been discussed since at least 1968, when a brief note was published to describe the use of a self-contained radiographic unit at Los Angeles County Harbor General Hospital [7]. The Faxitron model 304 instrument described in that note is a forerunner of instruments available today.

In our institution, specimen mammography has historically been available upon request via the Mallinckrodt Institute of Radiology, located on our campus. Under this paradigm, when a radiograph is required, a pathology resident or fellow makes an appointment (typically for the same day or next day), hand-carries the specimen to the mammography suite (7 min’ walk from pathology), and obtains the necessary image with the assistance of a radiology technician and radiologist. For core biopsy and mastectomy specimens, imaging has been performed upon tissue or paraffin blocks on an as-needed basis. For partial mastectomy (lumpectomy) specimens, an immediate postoperative radiograph is taken and provided to pathology. Nonetheless, the need for subsequent imaging could potentially arise, and would be handled in the same way as for other specimens.

In a busy surgical pathology laboratory, specimen radiography is a valuable tool, limited by availability of the imaging device. We hypothesized that greater access to this technique through in-lab radiography would result in improvements in turnaround time and reductions in paraffin block utilization. To test this hypothesis, we conducted a trial of in-laboratory cabinet radiography and determined whether this resource improved resource utilization in pathology.

## Methods

### Setting

The study was carried out in a College of American Pathologists-accredited, Clinical Laboratory Improvement Amendments-certified academic surgical pathology laboratory. The Institutional Review Board at Washington University determined that the study did not represent human subjects research (ID #201808076). Two breast specimen imaging modalities were used, as described below, and were compared to cases in which no imaging was performed. “Lumpectomy” cases included those described as partial mastectomy and needle-localization biopsy, while “mastectomy” cases included all total mastectomies (simple and modified radical).

### Baseline paradigm (“Specimen mammography”)

The baseline condition was specimen mammography performed in the breast imaging unit of the academic medical center. Under this paradigm, the images were examined by breast radiologists and any calcifications either circled on the radiograph or pointed out to the pathologist in the reading room. To quantify test utilization under this paradigm, we queried radiology records to retrieve the list of cases undergoing specimen mammography for the 12-month 2015 calendar year. Data retrieved included specimen type (core biopsy, lumpectomy, or mastectomy), turnaround time measured in integral working days from accession to signout (total number of Monday–Friday business days or portion thereof), and block utilization (for lumpectomy and mastectomy). Cases for which more than one specimen was radiographed were only counted once.

### In-laboratory radiography paradigm (“In-lab specimen imaging”)

A Faxitron PathVision cabinet radiography system was provided on a trial basis by the vendor (Faxitron Bioptics, LLC, Tucson, Ariz.). This instrument was installed in the gross pathology room and made available for use by pathologists, pathology trainees, and pathologists’ assistants. Images were recorded using bundled software and analyzed by pathologists. Radiography was performed systematically at the time of grossing for all core biopsies done for calcifications, and on an ad hoc basis for lumpectomies and mastectomies whenever deemed useful to identify a clip or lesion. We did not distinguish between radiographs taken of wet tissue versus blocked or embedded tissue.

To characterize test performance under this modality, we retrieved all cases accessioned during the 48-business-day trial period in April to July 2016. Data retrieved included specimen type (core biopsy, lumpectomy or mastectomy), turnaround time measured in integral working days from accession to signout, and block utilization (for lumpectomy and mastectomy). Cases for which more than one specimen was radiographed were counted once.

### Cases not requiring imaging (“No specimen imaging”)

To characterize breast pathology turnaround time when imaging was not required, we retrieved all consecutive breast cases from 1/1/17 to 3/15/17. This period was chosen because specific breast part types (core biopsy, lumpectomy and total mastectomy) began to be tracked as such within our laboratory information system on 1/1/17. Clinical records were reviewed to exclude any case taken for specimen mammography as part of pathologic examination.

### Pathologic evaluation of specimens

Outside of the differences in gross handling and imaging of the specimens as described above, all specimens were examined using standard H&E slides and any necessary ancillary immunostains under all three of the study paradigms.

### Statistics

Data on case volume, specimen type, gross pathology, turnaround time were extracted from the laboratory information system and analyzed using Excel 2011 (Microsoft Corp, Redmond, Wash.) and Prism 7 (GraphPad Software, San Diego, CA).

## Results

### In-lab imaging was used more frequently than specimen mammography during a comparable period

A Faxitron imaging cabinet was made available on a trial basis, and standard operating procedures were established to guide its use. Per this procedure, all core biopsies taken for calcifications were imaged, so that the calcifications could be placed in the first tissue block (or blocks, if too numerous to fit in one cassette). The procedure specified that lumpectomy and mastectomy specimens should be imaged as needed, e.g., to localize clips.

We documented that in-lab availability of specimen imaging increased utilization, as compared with a paradigm requiring the specimen to be transported outside the lab to the mammography suite. During the Faxitron trial period, the instrument was used 144 times, corresponding to 1107 instances if normalized to a full year. In comparison, specimen mammography in the radiology suite was sought by pathology 45 times in the 1-year comparison period (Table 1). Since core biopsies were imaged on Faxitron in a protocolized way (all cases imaged), it is trivial to observe that the Faxitron increased utilization for core biopsies. Much more relevant is the observation that use of the Faxitron led to increased imaging of resection specimens (increase from 0 to 115 lumpectomy specimens imaged on a per-year basis, and increase from 36 to 638 mastectomy specimens). Since those images were acquired only on an as-needed basis, we infer that there are many cases for which imaging would be desirable but for which its use is inhibited by lack of easy availability.

**Table 1:** Utilization of specimen radiography during the reference period, compared with Faxitron during trial period

	Core biopsy	Lumpectomy	Mastectomy	Total
*Cases taken to radiology*				
Actual in 1-year period	9	0*	36	45
*Cases imaged using Faxitron*				
Actual no. of cases in 48 days	46	15	83	144
Normalized to 1-year period	354	115	638	1107

*All lumpectomy specimens imaged by breast health center prior to delivery to pathology; no additional imaging requested or performed by pathology

### In-lab imaging reduces turnaround time of cases requiring imaging to the same as un-imaged cases

**Fig. 1:**
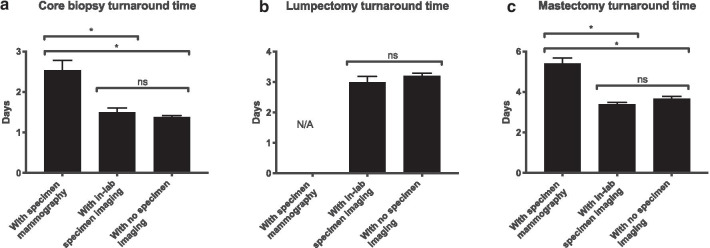
Turnaround time (TAT) of **a** core biopsy cases, **b** partial mastectomy (lumpectomy) cases, and **c** total mastectomy cases, measured in working days from case accession to signout. Radiology cases are those taken to radiology for imaging; Faxitron cases are those undergoing in-lab imaging during the trial period; unimaged cases are all cases processed during the reference interval, excluding those that were taken to radiology. *p < 0.05

Under the paradigm in which specimens are transported to radiology when they require imaging, the imaging causes delayed turnaround from accession to signout, as compared with cases not requiring imaging (Fig. 1). We documented this effect for core biopsies (mean difference 1.15 days, 95% CI 0.7–1.62, p < 0.0001 by one-way ANOVA with Bonferroni post-test) and mastectomies (mean difference 1.73 days, 95% CI 1.18–2.27), but not for lumpectomies, as none were imaged during pathology handling in 2015, possibly because at our institution these specimens are accompanied by a specimen radiograph by default (taken in radiology by staff from the breast health center prior to specimen accessioning). The delay attributed to imaging occurs because the imaging is typically done on the day after the request, and additional histologic leveling or block submission must then take place.

We hypothesized that in-lab availability of specimen radiography would eliminate the effect of imaging on turnaround time. Indeed, with Faxitron availability, the turnaround time of cases requiring imaging was not significantly higher than those processed without imaging during the reference period (Fig. 1), and this occurred for all three specimen types. For core biopsies, the difference between Faxitron cases and unimaged cases was 0.12 days (p = 0.86 by ANOVA with Bonferroni post-test); for lumpectomies, 0.21 days (p = 0.47 by two-tailed Student t-test); for mastectomies, − 0.28 days (p = 0.60 by ANOVA).

### In-lab imaging reduces block utilization in breast pathology

**Fig. 2:**
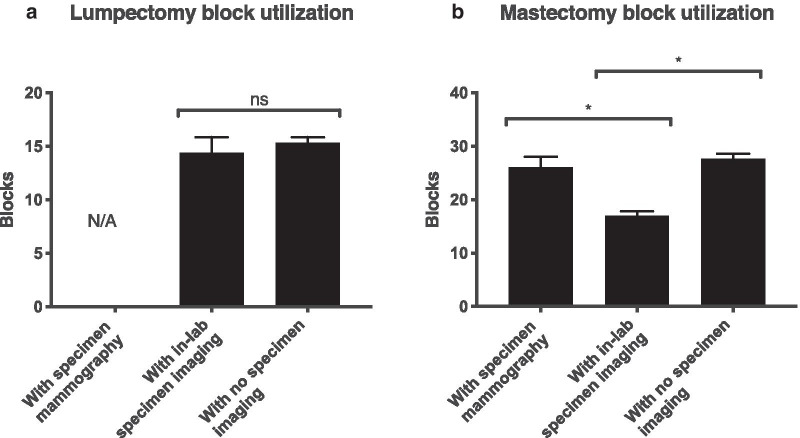
Block utilization in **a** lumpectomy and **b** mastectomy cases, on an individual specimen basis (main specimen only), for cases that were taken to radiology, for cases imaged on Faxitron, and for cases not taken to imaging. *p < 0.05

Breast excision specimens are typically submitted for imaging when initial pathologic examination (gross or microscopic) fails to demonstrate the features of interest, such as lesions and/or clips. We hypothesized that cases requiring imaging would require an increased number of tissue blocks (reflecting additional blocks embedded after imaging). This could not be substantiated for lumpectomy cases (as none were taken to radiology, Fig. 2a), and surprisingly, mastectomies requiring imaging in radiology did not result in embedding of significantly more blocks than those not taken for imaging (Fig. 2b, mean difference -1.6 blocks, 95% CI − 5.9 to 2.7, p > 0.9999 by one-way ANOVA with Bonferroni post-test).

As a corollary, we hypothesized that availability of the Faxitron would reduce block utilization in resection cases, since areas of interest (particularly clipped areas) could be identified and targeted preferentially for embedding. For lumpectomy cases, the Faxitron did not result in a lower number of blocks submitted as compared with unimaged cases (Fig. 2a, difference 0.94, 95% CI − 2.71 to 4.58; p = 0.61 by Student t-test), but for mastectomies, the Faxitron resulted in a striking decrease in block utilization (Fig. 2b, difference − 10.6 blocks, 95% CI − 14.7 to − 6.47; p < 0.0001 by one-way ANOVA with Bonferroni post-test).

## Discussion

We sought to determine the effects of in-lab imaging availability upon breast turnaround time and block utilization in the surgical pathology workflow.

The first major finding in this study is that in-lab imaging reduced the turnaround time of both core biopsy and mastectomy cases requiring imaging, to a level not significantly different from cases not requiring imaging. The TAT improved by 1.04 days for core biopsies and 2.01 days for mastectomies. This decrement appears clinically significant and will confer meaningful patient benefit. The improvement in TAT seen in our series is larger than that of 0.4 days described for mastectomies by Kallen et al. in their study of similar design [8].

Our second major finding is that when the in-lab imager was available, it reduced mastectomy block utilization by 10.6 blocks per imaged case. Back-of-the envelope calculations show that this reduction has beneficial downstream effects on histology workload. In a lab with 638 cases imaged per year, the predicted decrement in workload per year is 6800 blocks. Although the cost to process a single block will vary across laboratories, a 2017 analysis from our facility (unpublished data) indicated a cost of $11.62 to process and embed one block and cut and stain one H&E section. Under this assumption, total savings are estimated at $79,016 annually. As assumptions and costs will change in different laboratories, this informal analysis would need to be performed more precisely in laboratories considering purchase of a cabinet X-ray device, using local parameters. Our finding is similar to the mean 7.3 block decrease described by Kallen et al. with use of the Faxitron [8]. Additional savings are expected to be derived from more targeted use of deeper H&E levels to find calcifications in core biopsy material.

It must be noted that the cost savings ascribed to the use of the Faxitron or a comparable cabinet imager must be balanced against the cost of acquiring, installing and maintaining the instrument. The marginal cost of operating the instrument is zero, since there are no reagents or film costs, but the cost of service contracts over the life of the instrument must be considered.

Interestingly, while in-lab imaging was associated with decreased block utilization in mastectomy cases, mammography performed in the radiology suite did not show this effect. To explain this surprising finding, we speculate that when in-lab imaging is unavailable, prosectors may proactively submit more blocks than usual from these cases in an effort to preempt the need for mammography. When the Faxitron is available, they simply acquire the radiograph before prosection and embed the appropriate tissue at the time of initial grossing.

We did not retrieve data pertaining to the timing of the imaging relative to initial gross examination, and therefore cannot determine whether the benefits are due to “proactive” imaging (taking radiographs before initial block submission, so that the right areas can be embedded initially), or rather due to the shorter time needed for imaging, irrespective of the sequence of events.

We did not determine whether the operating characteristics of the Faxitron were different for cases involving calcifications, clips, or both. Given that these are present in a random mix in any real-world surgical pathology case volume, it is unlikely that results of such an analysis would be actionable.

We also did not determine whether the Faxitron permitted “better” pathologic examination, in the sense of reducing the number of cases where calcifications or clips are not found. Our assumption was that the vast majority of cases met the standard of care under both regimes, i.e., with exhaustive identification of calcifications and clips whenever possible.

Certain factors may reduce the utility of in-lab specimen radiography. A caveat of in-lab imaging is that the pathologist no longer has the special expertise of a radiologist at his or her immediate disposal, and calcifications may not be recognized, or potentially, findings may be overcalled as calcifications. However, in either case, histologic examination will be performed and will provide a gold standard. We did not provide any training to the pathologists and pathologists’ assistants in interpretation of Faxitron images. In addition to the uses we describe here, specimen mammography has been invoked for intraoperative margin assessment [9, 10]. It is not known whether a pathologist with or without special training could make these assessments in a reliable manner. Biopsy clips may migrate away from the site of initial placement due to the “accordion” effect that can occur when compression is released after biopsy [11]. In these cases, embedding the clip site may result in missing the tumor. Clips may also be lost due to intraoperative use of suction [11], or lost in the course of specimen opening and fixation. Specimen radiography makes it possible to rapidly ascertain the absence of a clip, but cannot prove whether the clip was lost or was not encompassed within the specimen. In some of these scenarios, histologic stigmata of an indwelling clip, such as giant cell reaction or clip-shaped cavities, may be identified histologically and serve to clarify the situation.

Advantages of the present study are that it quantitatively measures the benefit that can be obtained from a specific lab workflow improvement. As a study of consecutive cases, it is relatively unbiased. Compared to another study in this area [8], it has the benefit of including subgroup analyses for all three of the most common breast cancer-related specimen types: core biopsy, partial mastectomy, and total mastectomy. Disadvantages include the relatively short time period of the study intervention. It is not known whether there is a wash-in period for the effects of the Faxitron intervention. In this single-institution study, we have been able to make a point estimate of the benefit of in-lab imaging, but cannot estimate a confidence interval, which would help other labs realistically anticipate the gains that they may experience. We did not measure the effect of in-lab radiography on pathologist time utilization or pathologist morale, although anecdotally, both of these factors were much improved by availability of the Faxitron.

## Conclusion

In conclusion, in-laboratory breast specimen radiography allowed cases requiring imaging to be signed out in the same timeframe as cases not requiring imaging. For mastectomy cases, the availability of the Faxitron also reduced block utilization. These data present an opportunity for quality improvement in breast pathology, and may be useful to laboratories considering capital investments in similar instruments.

## Data Availability

The data are available from the corresponding author on reasonable request.
